# Some Remarks on Amoebic Dysentery

**Published:** 1908-11

**Authors:** Geo. M. Niles

**Affiliations:** Atlanta, Ga.


### SOME REMARKS ON AMOEBIC DYSENTERY.*

BY GEO. M. NILES, M. D., ATLANTA, GA.

The synonym of amoebic dysentery—tropical dysentery—would lead some to suppose it of small interest to physicians in temperate latitudes; but, since our flag has been planted in the Philippines, giving rise to a constant stream of travel to and fro; in addition, the Panama canal, bringing thousands of our people, who labor there, into intimate touch with us; and also the large and growing fleet of fruit vessels, constantly plying between our shores and the South—and Central—American countries, afford such facilities for the introduction of this disease, that this subject may at any moment be of interest to communities far removed from the tropics.

The writer recently saw four cases in New York City, besides learning of some cases occurring as far north as Maine.

Amoebic dysentery, sometimes called intestinal amobiasis, is a colitis, latent, subacute, acute or chronic, caused by the amoeba coli.

Liver abscess is so common an accompaniment as to be almost considered one of the lesions. Gilman Thompson reports thirteen fatal cases, ten of which had abscesses of the liver.

It is prevalent in tropical countries, and to some extent in the extreme southern states. It is much more frequent in cities than is generally supposed.

The source of infection is chiefly contaminated water, green vegetables or fruit. Musgrave has found the amoeba on dishes washed in tap water, on the surface of uncooked vegetables, such as lettuce, in milk, and on the hands of attendants. Selli found them growing six feet below the surface of the soil near dysenteric stools, and also at an elevation of 5,000 feet.

The amoeba coli is about five times the size of a red blood cell, colorless, or with a pale greenish hue; has granular contents, a faint renders and active psendopodia.

To obtain specimen for examination, flakes of mucus or pus should be selected; or the mucus could be obtained by passing a soft catheter, or through a rectal speculum. Preferably a saline cathartic should be given, as suggested by Musgrave, and the fluid portion of the stool examined. For satisfactory exam-

*Read before Fulton County Medical Society.

ination the bed-pan should be warmed, and brought to the laboratory immediately; or, if this is not practicable, the specimen should be kept warm until ready for examination, and then placed on a warm slide or stage. Several preparations shold be made, and carefully looked over for the amoeba or amoeba-like bodies.

Generally they will be found in motion, and can be recognized. Swollen and altered epithelial cells must be distinguished from them. This parasite is sometimes found in great numbers; again only a few may be present. The number present seems to bear little relations to the severity of the attack.

Should there be a liver abscess, the amoeba will probably be found in the pus along with the leucocytes and broken-down liver cells, though this is not always the case.

*Pathology.*—There is oedematous swelling of the intestinal surface and infiltration of the submucosa, followed by ulcers, chiefly in the coecum and flexures of the colon and rectum. These ulcers have thickened irregular openings, but which are small in comparison to the destroyed tissue underneath the mucosa. As this sloughs away, extensive irregular ulcers may appear; or sometimes the ulcers are connected by fistulous channels. Sometimes when the disease has been in progress for a while, in the intestine may be seen ulcers in different stages, as well as cicatrices of those already headed. Occasionally the cicatrices contract, causing either irregularity in the surface of the mucosa, or strictures of the gut.

Should the amoeba reach the liver, abscesses follow, which may be either so small as to look like translucent dots; or larger, containing, besides amoebae, fat cells, degenerating liver cells and red blood corpuscles; or still larger, having thick hemorrhagic walls of liver tissue. Sometimes there are necrotic areas in the liver, unaccompanied by abscess. Occasionally this process extends, causing necrosis and liquefaction of tissue without inflammatory reaction developed by pus bacteria. This effect Thompson believes to be caused by toxins developed by them within the intestine.

*Symptoms.*—Amoebic dysentery usually begins with a moderate and painless diarrhoea, alternating with short and irregular periods of constipation. During this period of constipation the patient feels fairly well. There is usually a slight fever, but seldom nausea or vomiting; while griping and tenesmus, if present at all, occurs only at the beginning of the disease.

The stools are at first copious, watery of a grayish color and, later on, mucous and bloody. They vary in number, sometimes running as high as forty in twenty-four hours. The patient becomes emaciated and and anaemic from the constant drain both of blood and albumen. The stools vary in appearance from day to day, but are all times fluid in consistency, and extremely offensive. Some cases described by H. A. West lasted for weeks without blood in the stools. In other cases symptoms of hepatic abscess with fever preceded the bowels manifestations.

The course of the disease generally runs through two or more months, followed by slow irregular convalescence. Relapses are frequent, arising upon the slightest indiscretion.

Prophylaxis is very important. All drinking water should be boiled, and dishes should be washed in boiled water, also the hands. Raw fruits or vegetables should first be put on ice, and then have boiling water poured over them. This will kill the amoeba. The vaginal douche or rectal enema from suspected water should be avoided. It is also important to sterilize the stools. This may be safely accomplished with carbolic acid 1 to 1,000. The same precautions should be observed with soiled linen.

*Treatment.*—In the acute form the patient should be put to bed, and on liquid diet, as barley-water, bouillon, broths, gruels, white of raw eggs, or peptonized milk diluted one half with either lime or barley-water. Either Sanatogen or Somatose will be found helpful, and are generally well borne. For the pain or colic in the abdomen use hot stupes, poultices, or hot-water bag.

*Internal Medication.*—Both Musgrave and Kemp object to bismuth preparations, in that they coat over the ulcers, thus interfering with local treatment. A possible exception to this is the subgallate of bismuth. In the initial stage cleanse the intestinal tract with sulphate of magnesia or castor oil. Some use calomel, but the writer is not much in favor of it in this stage of the disease. Neither has the writer gotten satisfactory results from the combination of salol guaiacol and ipecac so highly thought of by some obesrvers. Strong reports some benefit from acetozone 1 to 5,000, or 1 to 3,000 in carbonated water internally, 1 to 2 litres in twenty-four hours, in divided doses. Other valuable remedies are salicylate of guaiacol grs. v to x, given three or four times daily; tannalbin, grs. x; tannigen grs. x; tannigenaform grs v to x. Dilute hydrochloric acid with pepsin, or alone, is of value, for often the secretions of the stomach are in abeyance. Vomiting should be treated as that arising from any other gastric irritation.

Local treatment is very important for the acute, latent and chronic forms. The following are destructive to the amoeba as well as to other bacilli:

Acetozone 1 to 1,000. Most so.

Alphozone 1 to 1,000. Nearly as much.

Argyrol or protargol 1 to 500.

Bisulphate of quinine 1 to 500.

Thymol 1 to 2,500. Very good.

Permanganate of potash 1 to 2,000.

Peroxide of hydrogen 5 to 10 per cent.

Tuttle recommends cold water (45 F.) given in knee-chest position, and contained half an hour.

*Method of Irrigation.*—A glass irrigator with a colon tube with an opening preferably at the end should be used. If the ulcers are low down in the rectum, or there is extreme tenesmus, the physician may use an ordinary rectal tip.

When administering the irrigation, the foot of the bed should be elevated 12 to 18 inches, and the patient placed in Sims position, or with his hips elevated on the pan. As the irrigation proceeds, his position should be changed so the fluid will tend to gravitate into the caput coli. This is admirably described in Kemp's book on enteroclysis and hypodermoclysis. At least one to two litres should be injected, and should be retained 5 to 15 minutes—the longer the better. A smaller quantity may be used at the start, should much irritation be present.

The knee-elbow position may be used in chronic or latent cases, but will hardly prove feasible in the acute. Should the irrigation cause excessive pain, it may be preceded (half hour) by two ounces normal salt solution containing morphine 1-4th gr. and belladonna 10 drops. This should be done only once or twice in the first twenty-four hours of the treatment, and not repeated, if possible to avoid it.

Kemp sometitmes uses acetozone 1 to 5,000 combined with quinine 1 to 750, giving two to five enemas in twenty-four hours, according to the severity of the case. Should cold injections be badly borne, hot (120 F.) may be used instead. And should any of these preparations disagree, the list is broad enough to allow discretionary changes.

In latent cases bowels should be opened freely, and daily injections of quinine and acetozone given. Should large or small injection fail to be retained, recurrent irrigation (preferably with the Kemp tube) should be employed. One or two gallons of half-strength solution may be used, with the foot of the bed considerably elevated. The tannic acid preparations suggested in acute cases, are admissible; also the same injections, but not so often.

Change of climate is frequently of much benefit.

High fever should be controlled by sponging, antipyretics not being often indicated. For weak heart strychnine or spartein may be used, as in cardiac weakness from any other cause.

Experiments are being made as to the efficacy of giving 1-4 gr. of fluorescin in 6 ozs. of water, and placing the patient in full electric light. This promises well in latent or chronic cases, but can hardly, as yet be intelligently reported on.

The writer wishes to make acknowledgements to Messrs. Musgrave, Strong, Gilmore Thompson, Boardman Reed, and especially to Dr. Robert Coleman Kemp, of New York, in whose gastro-intestinal service at Ward's Island and West Side German Dispensary, he was afforded facilities for studying this interesting and important disease.

408-9 Candler Building.

